# Wave Impedance Determination for PCB SIWs Considering Metal Roughness Loss Effects

**DOI:** 10.3390/mi15070883

**Published:** 2024-07-04

**Authors:** María Teresa Serrano-Serrano, Reydezel Torres-Torres

**Affiliations:** Instituto Nacional de Astrofísica, Óptica y Electrónica (INAOE), San Andrés Cholula 72840, PUE, Mexico; tere_17abril@hotmail.com

**Keywords:** conductor surface roughness, substrate integrated waveguide, wave impedance, characteristic impedance

## Abstract

This paper presents an expression to determine the complex wave impedance of a substrate-integrated waveguide for the dominant TE_10_ propagation mode, notably enhancing the accuracy in modeling the corresponding imaginary part. This was accomplished by systematically identifying the need to consider additional conductor losses caused by the interaction of the propagating fields with the conductor material. In fact, by using the proposed expression, the complex impedance can straightforwardly be determined by combining propagation constant data, and the resistance that represents the loss associated with longitudinal currents occurring at the top and bottom walls, which are influenced by the conductor surface roughness. This allows for completely describing the characteristics of the waveguide when assuming uniform propagation along its length. Furthermore, since the voltage–current, the power–current, and the power–voltage impedances can easily be obtained from the wave impedance, the proposal enables representing the matching characteristics of the waveguide for circuit design purposes. An agreement between simulated and experimental insertion loss is achieved for substrate-integrated waveguides of two different widths when reconstructing the corresponding *S*-parameters using the determined wave impedance.

## 1. Introduction

Substrate-integrated waveguides (SIWs) are widely used in high-speed electronics due to their compatibility with printed circuit boards (PCBs) [1,2,3,4]. In fact, the corresponding structure and performance resemble those of a rectangular waveguide (RWG), enabling their application from interconnects [5,6] to resonators used for determining material properties [7,8,9] in microwave circuitry. In this regard, the full characterization of a uniform section of SIW propagating signals in the single mode can be achieved once the complex propagation constant (*γ*) and the wave impedance (*Z*_wave_) are known. Furthermore, from these parameters, the properties of the constituting dielectric and conductor materials can be inferred [10], the effect of the SIW when used as an access component can be de-embedded [11], and even equivalent circuit models can be implemented to represent these structures in practical circuits [12,13].

For the purpose of carrying out the abovementioned characterization, it is a common practice to obtain the attenuation (*α*) and phase delay (*β*) versus frequency (*f*) curves by applying the eigensolution of the thru-reflect-line (TRL) algorithm to data measured to SIWs varying only in length [10], which is simple and accurate. Conversely, obtaining *Z*_wave_ is problematic due to the significant effect of the signal launchers on the experimental return loss, even after de-embedding. Alternatively, treating the SIW as a RWG with effective dimensions [14] and assuming propagation in the dominant TE_10_ mode, *Z*_wave_ can be indirectly obtained from *γ* considering that the propagation occurs within a non-magnetic medium characterized by the permeability of vacuum [15]. Notwithstanding, since the conductor walls of the SIW are made of copper, the corresponding losses at microwave frequencies are noticeable and accentuated by the skin effect; thus, it is inferred that an effective complex permeability needs to be considered, rather than *μ*_0_, when calculating *Z*_wave_.

For the case of RWGs, a modeling approach to account for the losses occurring within the conductor material was proposed in [16] for obtaining *Z*_wave_, which provides good results for hollow waveguides. Evidently, the lossless dielectric assumption required to simplify the corresponding analysis yields substantial errors for SIWs constructed, even with low-loss PCB dielectric materials. Therefore, to take into account both dielectric and conductor losses, a transmission line representation can be employed to indirectly determine *Z*_wave_ [12]. Bear in mind, however, that in current technologies, the copper layers forming the top and bottom walls are intentionally roughened to promote adherence with the dielectric laminate [6]. Therefore, it is essential to account for the increase in loss introduced by the rough conductor surface. In this regard, knowledge of the surface topography eases the model’s implementation [17]. Nevertheless, we demonstrate that using the nominal rms roughness (*R_q_*) specified by the PCB manufacturer provides acceptable accuracy for characterizing SIWs operating at tens of gigahertz, and avoids the additional inspection of the conductor surfaces, either before or after fabrication.

Here, *Z*_wave_ is obtained by combining experimental *γ* data and a parameter extraction for the conductor losses, including the effect of the roughness of the metal foils used to form the waveguide. In fact, we demonstrate that, even though the real part of *Z*_wave_ can be computed from *γ* using the traditional approach [15], its imaginary part is strongly sensitive to losses occurring within the conductor material due to the time-varying fields. This sensitivity underscores the need for improvement in determining Im(Z_wave_) to ensure accuracy in characterizing SIWs operating at microwave frequencies. For this reason, a new expression explicitly incorporating the resistance of the top and bottom waveguide walls for calculating *Z*_wave_ is presented in this paper, along with the corresponding implementation for experimentally analyzing prototypes in practical PCB technologies. Moreover, since the increase in the resistance with frequency is substantial when these walls are rough, this effect is also considered during the development of the proposal.

Once *Z*_wave_ is determined, it can be used to analyze the intrinsic properties of the waveguide propagating in the single TE_10_ mode in a similar manner, as the characteristic impedance concept is associated with transmission lines operating in the quasi-transverse electromagnetic mode. Moreover, the power–current, voltage–current, and power–voltage impedances (i.e., *Z*_vi_, *Z*_pi_, and *Z*_pv_, respectively) can directly be obtained depending on the desired description for the SIW [18]. Additionally, although the resistance associated with the top and bottom walls is demonstrated to significantly impact the response of PCB SIWs, it is also observed that the effect of the corresponding internal inductance is negligible. This enables the determination of the complex relative dielectric permittivity (${\hat{\varepsilon }}_{r}$) and, consequently, the loss tangent (tan*δ*) of the laminate used as the PCB substrate within the measurement frequency range.

## 2. Relationship between *Z*_wave_ and *γ* in PCB SIWs

**Figure 1:**
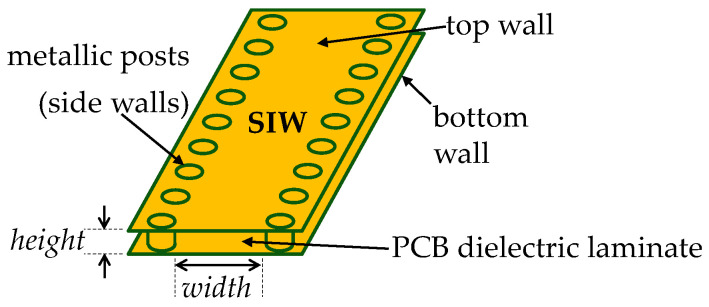
Sketch showing the structure of a PCB substrate-integrated waveguide.

**Figure 2:**
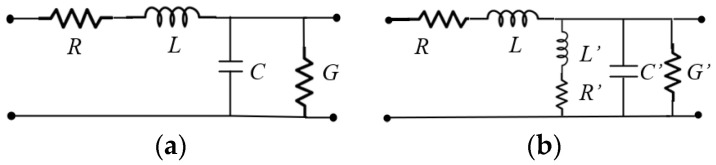
RLGC model for a RWG: (**a**) generic transmission line model and (**b**) modified topology reported in [12].

Consider the sketch shown in Figure 1, where a simplified representation of a PCB SIW is presented. This structure can be assumed to behave as a RWG, provided that the adjacent metallic posts forming the side walls exhibit a separation much smaller than the wavelength of the propagating signals [10], which can easily be achieved using commercial PCB fabrication processes and for circuit operation at microwave frequencies. Hence, this section is dedicated to establishing a relationship that allows for the determination of *Z*_wave_ from *γ*, assuming that the single TE_10_ propagation mode as in a RWG is taking place. In this case, *Z*_wave_ is usually calculated from the following simplified expression [15]:

(1) $$Z_{wave}=j\frac{\omega \mu_{0}}{\gamma }$$

where *j*^2^ = −1, *ω* = 2π*f* is the angular frequency and *μ*_0_ is the permeability of vacuum. To distinguish the real and imaginary parts of *Z*_wave_, Equation (1) is expanded to yield:

(2) $$Re(Z_{wave})=\frac{\omega \mu_{0}}{\beta }$$

and

(3) $$Im(Z_{wave})=\frac{\omega \mu_{0}\alpha }{\beta^{2}}$$

where *γ* = *α* + *jβ* was used, and *α*^2^ ≪ *β*^2^ was assumed, given that the phase delay significantly outweighs the attenuation in practical scenarios. Notice in these expressions that the magnetic field is assumed to be propagating through a vacuum, hence, $\mu_{0}$ is used. To circumvent this inaccuracy when the interaction of the magnetic field with the conductor material causes loss, the well-known RLGC transmission line model in Figure 2a is useful for analyzing the waveguide [16], which can be modified into the equivalent representation shown in Figure 2b [12]. Notice in these figures that, since the per-unit-length series elements *R* and *L* exhibit no variation between the two models, *Z*_wave_ can be expressed as for a generic transmission line by [19]:

(4) $$Z_{wave}=\frac{R+j\omega L}{\gamma }$$

It is important to notice that Equation (4) becomes Equation (1) in case *R* = 0 and *L* = *μ*_0_ are assumed, which is equivalent to neglecting the loss and delay associated with longitudinal currents occurring on the top and bottom walls of the SIW. To assess the impact of neglecting these effects, here, an expression for the complex *Z*_wave_ is developed, considering *R* > 0 and *L* the total series inductance of the waveguide. This inductance is provided by the sum of the inductances accounting for the interaction of the magnetic field with the non-magnetic media external to the conductor material (i.e., represented by *μ*_0_) and internal to it (i.e., *L*_int_). For this purpose, several assumptions are made. For instance, although α>0 should be considered for SIWs, in PCB technology, these waveguides are made of low-loss dielectric materials, and copper. Hence, α≪β at microwave frequencies. In this case, after substituting *L* = *μ*_0_ + *L*_int_ and *γ* = *α* + *jβ* into Equation (4), it is possible to obtain:

(5) $$Re\left(Z_{wave}\right)\approx \frac{\omega \beta {(\mu }_{0}+L_{int})+\alpha R}{\beta^{2}}$$

and

(6) $$Im(Z_{wave})\approx \frac{\omega \alpha {(\mu }_{0}+L_{int})-\beta R}{\beta^{2}}$$

Bear in mind that *R* and *L* are series elements in the transmission line model and thus are associated with longitudinal currents, which only take place in the top and bottom walls of an SIW operating in the TE_10_ mode. Therefore, to quantify the impacts of *R* and *L* in *Z*_wave_ through Equations (5) and (6), firstly, the surface impedance (*Z*_S_) for a smooth conductor is expressed as [20]:

(7) $$Z_{S}=R_{smooth}(1+j)$$

In this equation, the resistance under a smooth-conductor assumption (*R*_smooth_) can be calculated from the skin depth (*δ*), the conductor conductivity (*σ*), and the height (*h*) of the waveguide in the direction parallel to the electric field by means of [12]:

(8) $$R_{smooth}=\frac{2}{\delta \sigma h}$$

where the numerator is multiplied by 2 to consider the resistance of the top and bottom walls, and *δ* is given by:

(9) $$\delta =\sqrt{\frac{2}{\omega \sigma \mu_{0}}}$$

**Figure 3:**
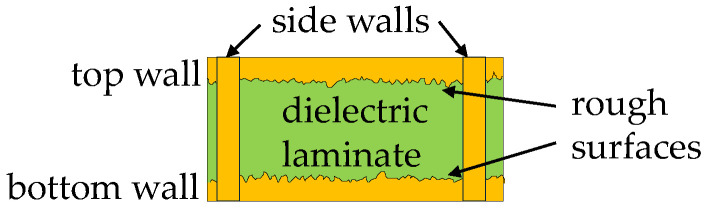
Simplified sketch of the cross-section of a PCB SIW illustrating the rough surfaces exhibited by the top and bottom walls.

Now, consider that the top and bottom walls of the SIW exhibit roughness, as illustrated in Figure 3. In this case, the imperfect surface introduces additional wave scattering that increases the loss experienced by a signal propagating along the waveguide. For incorporating this effect into the surface impedance model, the complex and frequency-dependent roughness factor $({\hat{k}}_{rough}=k_{1}+jk_{2}$) is combined with Equation (7) to write [21]:

(10) $$Z_{S\_rough}={\hat{k}}_{rough}Z_{S}=\left(k_{1}+jk_{2}\right)R_{smooth}\left(1+j\right)$$

From Equation (10), considering that the surfaces of the top and bottom walls are rough, *R* and $L_{int}$ can respectively be expressed as:

(11) $$R=Re(Z_{S\_rough})=K_{R}R_{smooth}$$

and

(12) $$L_{int}=\frac{Im(Z_{S\_rough})}{\omega }=K_{L}\frac{R_{smooth}}{\omega }$$

where $K_{R}=k_{1}-k_{2}$ and $K_{L}=k_{1}+k_{2}$.

To continue with the analysis corresponding with the wave impedance, substituting Equations (11) and (12) into (5) and (6) and making some simplifications yield:

(13) $$Re\left(Z_{wave}\right)\approx \frac{\omega \mu_{0}+K_{L}R_{smooth}}{\beta }$$

and

(14) $$Im(Z_{wave})\approx \frac{\alpha {(\omega \mu }_{0}+K_{L}R_{smooth})-\beta K_{R}R_{smooth}}{\beta^{2}}$$

In these equations, $(\alpha K_{R}+\beta K_{L})\approx \beta K_{L}$ was assumed, since not only α≪β, but also $K_{L}$ is a few times larger than $K_{R}$ for typical copper foils on PCB technology [22].

**Figure 4:**
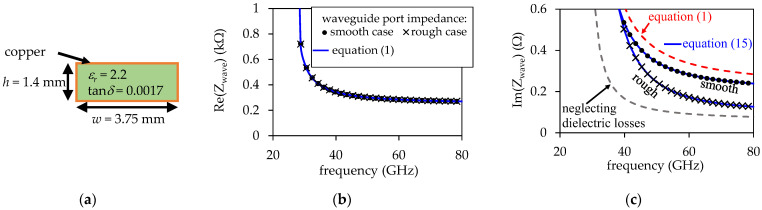
$Z_{wave}$ obtained from EM simulated data for a RWG assuming PCB materials considering smooth and rough conductor conditions: (**a**) cross-section of the simulated structure, (**b**) real part, and (**c**) imaginary part.

In order to verify the validity of the presented analysis, a RWG uniform along its length and exhibiting the cross-section depicted in Figure 4a was simulated using the commercial full-wave electromagnetic (EM) solver Ansys HFSS, employing the modal solution. In this regard, when establishing the simulation conditions for the RWG, waveguide ports were defined at the terminations of the structure. The material properties were specified for the filling dielectric, as depicted in Figure 4a, while copper was assumed to constitute the walls. The simulation covers the frequency range from 25 GHz to 80 GHz with a discrete sweep (i.e., without interpolations). Additionally, the dimensions of the waveguide are set to achieve a cutoff frequency of the TE_10_ mode, *f*_TE10_ ≈ 27 GHz. Two cases were simulated, one considering that all the inner surfaces of the walls are smooth, and another where the top and bottom walls exhibit a standard profile encountered in PCB technology. For this latter case, Huray’s causal model was defined to represent the surface impedance during the EM simulation by establishing a nodule radius *n_r_* = 0.5 μm and the Hall–Huray surface ratio *SR* = 3 [23]. In these simulations, the complex *Z*_wave_ was directly obtained from one of the excitation waveguide ports.

As illustrated in Figure 4b, no noticeable change was observed in the simulated Re(*Z*_wave_) curves with and without considering the conductor surface roughness, which suggests that the effect of the internal inductance for this structure is negligible for PCB materials and within this frequency range. This is expected since *L*_int_ significantly decreases with frequency, as expressed in Equation (12). Hence, including the internal inductance term in Equation (13) is necessary only at frequencies where the skin effect allows the current to flow within a significant portion of the cross-section of the top and bottom walls. Considering copper, this occurs well below 10 GHz. In this case, SIWs would need to be excessively wide to sufficiently lower their cutoff frequency, rendering them impractical for PCB applications. Hence, ${\omega \mu }_{0}\gg K_{L}R_{smooth}$ can be assumed in Equation (13), making it possible to apply Equation (2) for obtaining Re(*Z*_wave_) with accuracy in PCB SIWs operating at tens of gigahertz, as also shown in Figure 4b. Conversely, even when omitting the term $K_{L}R_{smooth}$ in Equation (14), the resulting expression for the imaginary part of the wave impedance differs from Equation (3); this is:

(15) $$Im(Z_{wave})\approx \frac{\omega \alpha \mu_{0}-\beta R}{\beta^{2}}$$

where *R* is defined in Equation (11). This is the novel definition of Im(*Z*_wave_) reported in this paper. In fact, Figure 4c illustrates that using the simulated *α*, *β*, and *R* and applying Equation (15) reproduces, with accuracy, Im(*Z*_wave_) for the smooth and rough cases. Conversely, when comparing the imaginary part of the simulated *Z*_wave_ with the calculation using Equation (1), significant discrepancy is observed in Figure 4c. Also note that, in this figure, determining *Z*_wave_ from the simulated *γ* and involving the effective dimensions of the waveguide, as in [16], is not appropriate as the dielectric losses are assumed to be negligible for the corresponding formulation to remain valid. In fact, the approach in [16] is more suitable for hollow waveguides.

Therefore, as explained in this section, accounting for the conductor effects in the determination of Im(*Z*_wave_) requires considering the βR term as proposed in this work. As *β* = Im(*γ*) can be easily extracted from experiments, the only unknown in Equation (15) is *R*. A simple strategy to obtain this parameter is explained hereafter.

## 3. Experimental Details

**Figure 5:**
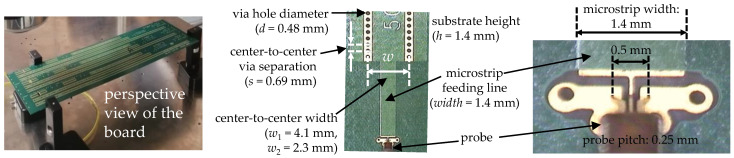
Photographs showing a perspective view of the prototype, the details of the microstrip-to-SIW transition, and a zoomed-in image of the probing pads.

Several SIW structures were built on the PCB prototype shown in Figure 5. The dielectric laminates used in this board exhibited a thickness, *h* = 1.4 mm, and nominal relative permittivity $\varepsilon_{r}^{\prime }=2.2$ and loss tangent tan*δ* = 0.0009 at 10 GHz [24]. On the other hand, the foils forming the top and bottom walls of the SIWs are made of low-profile copper with a root-mean-square surface roughness, *R_q_* = 1.198 μm ± 0.053 μm, as specified by the PCB manufacturer. The sidewalls are implemented using arrays of copper via holes, as described in Figure 5. Now, considering these dimensions and equivalences with a RWG [25], from the transverse center-to-center width *w* = 4.1 mm, a cutoff frequency *f*_TE10_ ≈ 27 GHz was obtained, whereas for *w* = 2.3 mm, the result is *f*_TE10_ ≈ 47 GHz. Since these SIWs are constructed on the same board layer, the propagating waves can be assumed to experience identical properties of the dielectric and conductor media. Thus, including SIWs of different widths within the prototype provides a way to experimentally verify consistency in the parameter extraction methodology for structures exhibiting different electrical responses. The microstrip lines used to apply the signals to the SIWs are shown in Figure 5. These lines were designed to excite odd electric modes and feature coplanar pad terminations for landing probes with a pitch of 250 µm. This allows for measuring *S*-parameters up to 80 GHz using a vector network analyzer (VNA), which was calibrated to shift the measurement plane up to the end of the probes through a line–reflect–reflect–match (LRRM) algorithm.

Since the core of the methodology is using experimental *γ* to characterize the uniform section of an SIW, these data are obtained free of the effect of the signal launchers (i.e., including the microstrip sections) using a line–line method based on the TRL formulation [26]. For this purpose, SIWs of two lengths, *l*_1_ = 76.2 mm and *l*_2_ = 254 mm, were implemented in the prototype for each width.

## 4. Parameter Determination

Electromagnetic waves propagate through dielectric and conductor media within SIWs and RWGs; thus, the overall energy loss and delay involve the interaction of waves with these two types of materials. Consequently, a simultaneous determination of model parameters for the effects associated with these interactions is necessary to assess their impact on the waveguide performance. In this regard, although obtaining the dielectric complex permittivity is not necessary to implement the model for *Z*_wave_ proposed in this paper, it is nonetheless included as the first step of the parameter-extraction methodology. This allows for presenting a complete modeling approach for a uniform section of SIW and facilitates comparing the dielectric and conductor-related losses occurring at microwave frequencies.

As demonstrated in Section 2, the effect of the internal inductance on the performance of the SIW can be neglected for PCB SIWs operating at tens of gigahertz; thus, since *β* = Im(*γ*) is already known from experiments, the relative permittivity $\varepsilon_{r}^{\prime }$ = Re(${\hat{\varepsilon }}_{r}$) can be obtained from [27]:

(16) $$\varepsilon_{r}^{\prime }=\frac{\beta^{2}+{\left(\frac{\pi }{w_{eff}}\right)}^{2}}{\omega^{2}\mu_{0}\varepsilon_{0}}$$

where $w_{eff}=w-d^{2}/0.95s$ is the SIW effective width [10], $\varepsilon_{0}$ is the vacuum permittivity, and *d* and *s* are defined in Figure 5.

**Figure 6:**
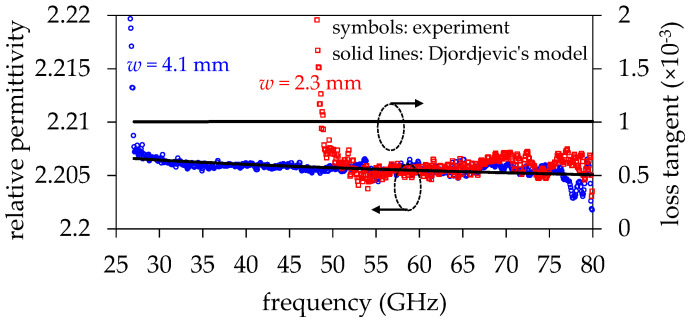
Permittivity and loss tangent determination using a causal model.

For PCB laminates, Djordjevic’s model can be used to consider the causal relationship between $\varepsilon_{r}^{\prime }$ and tan*δ* [28]. In the corresponding mathematical representation, $\varepsilon_{r}^{\prime }$ is assumed to exhibit a variation ∆ε′ between a lower and an upper frequency limit, $\omega_{1}$ and $\omega_{2}$, respectively. Furthermore, a useful simplification can be made without significantly losing accuracy at frequencies where $\omega \gg \omega_{1}$ and $\omega \ll \omega_{2}$. For the real part of the permittivity, the approximation is as follows [28]:

(17) $$\varepsilon_{r}^{\prime }\approx \varepsilon_{\infty }^{\prime }+\frac{∆\varepsilon \prime }{m_{2}-m_{1}}\frac{ln\left(\frac{\omega_{2}}{\omega }\right)}{ln10}$$

where $\varepsilon_{\infty }^{\prime }$ is the asymptotic value toward which $\varepsilon_{r}^{\prime }$ approaches at high frequencies, whereas $m_{1}=log10(\omega_{1})$ and $m_{2}=log10(\omega_{2})$. For the case studied here, the intention is representing $\varepsilon_{r}^{\prime }$ well above the cutoff frequency of the TE_10_ mode for the SIW with *w*_1_ = 4.1 mm, and below the cutoff frequency of the TE_30_ mode; this allows for assuming single-mode operation. In round numbers, this range is defined from 30 GHz to 80 GHz. Hence, for Equation (17) to remain valid, $\omega_{1}$ = 10^4^ rad/s and $\omega_{2}$ = 10^12^ rad/s are arbitrarily defined. Figure 6 illustrates the excellent model–experiment correlation for the relative permittivity for the SIW with *w*_1_ = 4.1 mm, where $\varepsilon_{\infty }$= 2.204 and $∆\varepsilon^{\prime }$ = 0.026 were obtained through curve fitting. Furthermore, Figure 6 also shows that, even though the model was implemented from data corresponding to one of the SIW widths, the $\varepsilon_{r}^{\prime }$ curves for the two structures with different widths barely differed from each other well above the cutoff frequency of their fundamental modes. This verifies the usefulness of using more than one SIW for obtaining experimental broadband relative permittivity data.

Now, employing Equation (17) under the same assumptions explained in [28], the loss tangent is approximately given by:

(18) $$tan\delta \approx \frac{\pi }{2}\cdot \frac{∆\varepsilon \prime }{ln(10)(m_{2}-m_{1})\varepsilon_{r}^{\prime }}$$

which allows for determining tan*δ* ≈ 0.001 as shown in Figure 6.

The next step is to calculate *R*, which is associated with the longitudinal currents taking place along the top and bottom walls of the SIW and thus suffers from the additional loss introduced by the surface roughness effect on these walls [29]. In this regard, *R*, can be obtained using Equation (11), which requires the knowledge of the roughness correction factor [30]. Owing to its simplicity and ability to maintain accuracy for low-profile copper foils, the mathematical form presented in reference [31] for *K_R_* is utilized here. This model assumes that a pyramidal array of spheres of fixed radius *r* allows for defining a unit cell for representing the surface roughness, and is expressed as:

(19) $$K_{R}=1+\frac{\;(\Delta K-1)c_{x}f}{c_{x}f+\sqrt{2c_{x}f}+1}$$

In this equation, $c_{x}=2\pi \mu_{0}\sigma r^{2}$, and *σ* is assumed here to be that of copper, *σ* ≈ 5.8 × 10^7^ S/m. Furthermore, considering an array of 14 spheres, Δ*K* = 8.33 and *r* ≈ *R_q_*/4.8 [32]; thus, *r* ≈ 0.25 μm is obtained by rounding *R_q_* = 1.2 μm from the PCB manufacturer’s specification detailed in Section 3.

**Figure 7:**
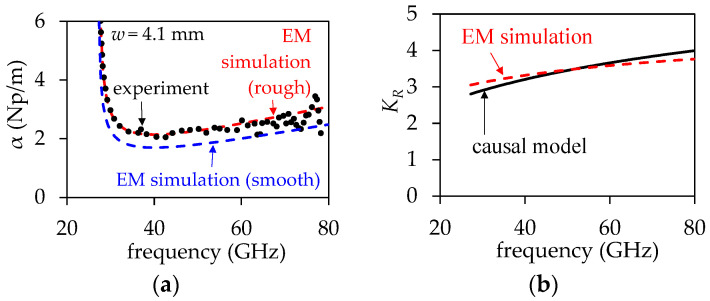
Verification of suitability of using Equation (20): (**a**) α from an EM simulation correlated with experimental data. (**b**) Comparison between *K_R_* = *R*/*R*_smooth_ obtained from the correlated simulation and from the causal model expressed by Equation (19).

To verify the suitability of using Equation (19), a 3D model of a uniform section of the SIW with *w*_1_ = 4.1 mm was simulated in Ansys HFSS. The simulation setup was defined as described in Section 2. However, in this case, the sidewalls were modeled using metallic posts, consistent with the fabricated structure shown in Figure 5. Moreover, the frequency-dependent permittivity and loss tangent of the dielectric material were defined based on the results shown in Figure 6. Afterward, a model–experiment correlation was performed for the attenuation curve by only varying the surface impedance on the top and bottom walls in the 3D model. The result of this correlation is shown in Figure 7a, demonstrating excellent agreement up to approximately 60 GHz. Beyond this frequency, the trend is well-predicted by the EM model, but some data dispersion is observed. This is attributed to the increased difficulty in accurately determining *α* at higher frequencies, even after rigorous de-embedding of the signal launchers, which involves multiple transitions. From this result, it is possible to illustrate in Figure 7b the concordance between *K_R_* obtained from the simulation and calculated using Equation (19). Hence, using the nominal value of *R_q_* provided by the manufacturer enables a very approximate calculation of *K_R_*, useful for the purposes of this paper. This fact is further corroborated in the Results Section.

## 5. Results

**Figure 8:**
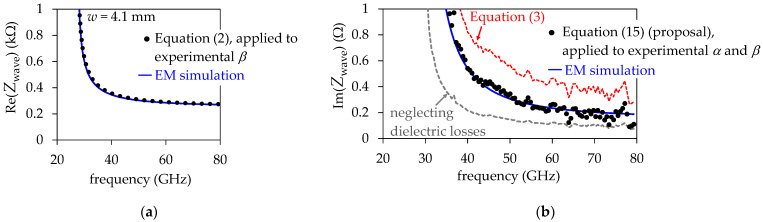
*Z*_wave_ curves calculated from experimental α and *β* data using different approaches. EM simulations accounting for the surface roughness effect on the SIW top and bottom walls are also depicted for comparison: (**a**) real part, and (**b**) imaginary part. The curves correspond to the SIW with *w* = 4.1 mm.

**Figure 9:**
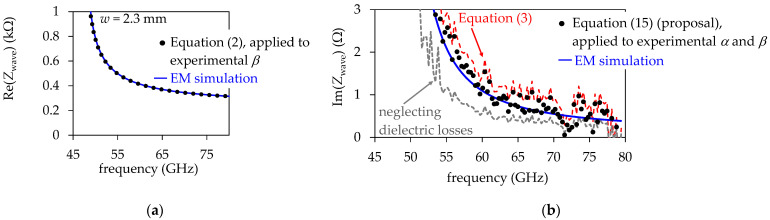
*Z*_wave_ curves calculated from experimental α and *β* data using different approaches. EM simulations accounting for the surface roughness effect on the SIW top and bottom walls are also depicted for comparison: (**a**) real part, and (**b**) imaginary part. The curves correspond to the SIW with *w* = 2.3 mm.

Figure 8a shows the Re(*Z*_wave_) versus frequency curve obtained from an EM simulation including the surface roughness effect for the prototyped SIW with *w* = 4.1 mm. This simulation is accurately reproduced by the calculation performed using Equation (2) and the experimental *β*. As previously mentioned, at frequencies of gigahertz, the inductance increase introduced by the surface roughness effect is negligible for practical PCB SIWs, and thus no correction in this equation is necessary. Nonetheless, in Figure 8b, it is observed that Equation (3) fails representing Im(*Z*_wave_), since the losses associated with the longitudinal currents occurring in the top and bottom conductor walls are neglected, underestimating the SIW’s overall loss. Likewise, as expected, the curve corresponding to neglecting the dielectric losses to determine Im(*Z*_wave_) from the experimental *γ* using the approach in [16] shows a significant discrepancy with the EM simulation. Conversely, the proposed Equation (15) and the parameter extraction strategy that led to its implementation allow to obtain a curve that correlates the EM simulation with accuracy. Complementary, Figure 9a shows the Re(*Z*_wave_) curves corresponding to the SIW that exhibits a higher cutoff frequency for the TE_10_ mode. For this structure, bear in mind that its reduced cross-section introduces higher insertion loss, which contributes to increasing the measurement uncertainty for loss-related effects. In this case, despite the data dispersion introduced by involving the experimental α for the calculation of Im(*Z*_wave_), there is a noticeable downward shift in the corresponding curve when considering the impact of *R* on the performance of this narrower SIW; this is illustrated in Figure 9b.

**Figure 10:**
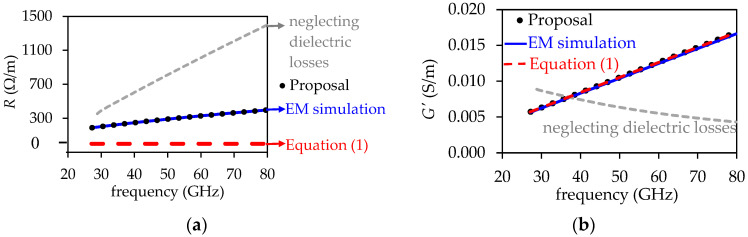
Curves for the circuit elements associated with the higher losses in the model shown in Figure 2b: (**a**) *R*, and (**b**) *G*’. The cases where *Z*_wave_ was calculated with simplified approaches are also shown.

To demonstrate the application of the proposed model for *Z*_wave_, the curves for the equivalent circuit elements in Figure 2b are obtained versus frequency using the following equations:

(20) $$R=Re(\gamma Z_{wave})$$

(21) $$L=\frac{Im(\gamma Z_{wave})}{\omega }$$

(22) $$G\prime =\omega \varepsilon_{0}\varepsilon_{r}^{\prime }tan\delta$$

(23) $$C\prime =\varepsilon_{0}\varepsilon_{r}^{\prime }$$

(24) $$Y_{RL}=\frac{\gamma }{Z_{wave}}-G^{\prime }-j\omega C\prime$$

(25) $$R\prime =Re\left(\frac{1}{Y_{RL}}\right)$$

(26) $$L\prime =\frac{1}{\omega }Im\left(\frac{1}{Y_{RL}}\right)$$

where $Y_{RL}$ is the shunt admittance associated with the series connection of R′ and L′. Thus, when using the experimentally determined data for *γ*, $\varepsilon_{r}^{\prime }$, and tan*δ* in the previous equations, the curves shown in Figure 10 are obtained for the R and G′ elements, which represent the more significant losses occurring in the SIW. A correlation is observed between the experimentally determined data and EM simulations performed using the 3D model for the SIW. In contrast, unexpected curve trends for R and G′ are obtained when neglecting the dielectric losses in the calculation of *Z*_wave_: R is clearly overestimated while G′ drops with frequency. This can be seen in Figure 10a,b, respectively. Likewise, ignoring the longitudinal currents in the top and bottom walls when obtaining this impedance as in Equation (1) yields R = 0, whereas R′, which is predicted to be negligible by the EM simulation, is overestimated.

Finally, the *S*-parameters of an SIW were reconstructed using the experimental *γ* and *Z*_wave_ obtained from Equations (3) and (15). To do so, the following transmission line- based equation was employed to firstly obtain the *ABCD* parameters of a uniform section of SIW of length (*l*):

(27) $$T=\left[\begin{array}{cc} cosh(\gamma l) & Z_{wave}sinh(\gamma l)\\ Z_{wave}^{-1}sinh(\gamma l) & cosh(\gamma l) \end{array}\right]$$

**Figure 11:**
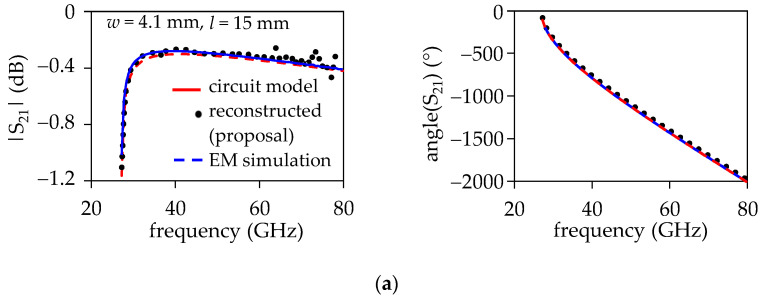

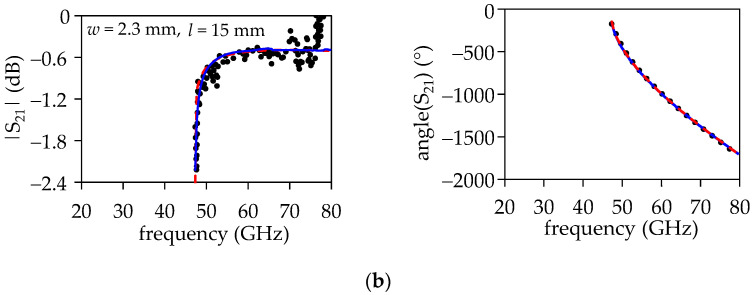
Model–experiment correlation for the insertion loss obtained through a reconstruction of *S*-parameters using *γ* and *Z*_wave_ for the SIW with: (**a**) *w* = 4.1 mm and (**b**) *w* = 2.3 mm; *l* = 15 mm was assumed for both cases. For the calculation of *S*_21_, the *S*-parameters are referenced to *Z*_wave_ for minimizing the return loss.

Afterward, an *ABCD*-to-*S*-parameter transformation is performed by considering *Z*_wave_ as the reference impedance. This implies that perfect matching is assumed at the SIW terminations, and the signal transmission is maximized. Hence, the model can be directly assessed by analyzing the insertion loss (i.e., |*S*_21_|). Figure 11a,b show that the reconstructed magnitude and phase for *S*_21_ corresponding to the uniform section of the SIW agree with the EM simulations for the two considered widths. Furthermore, considering the experimental *γ* and *Z*_wave_ obtained through applying the proposal, it is possible to implement the equivalent circuit for the SIW using Equations (20)–(26). This enables the representation of the insertion loss of the SIW in a circuit simulator. The corresponding curves are also shown in Figure 11a,b. This verifies the usefulness of the proposal for obtaining *Z*_wave_, which can also be converted to other impedance definitions (i.e., *Z*_pi_, *Z*_vi_, and *Z*_pv_) [18,33].

## 6. Conclusions

An expression for obtaining the complex wave impedance of an SIW operating in the TE_10_ propagation mode was proposed. It was shown that the effect of the conductor surface roughness exhibited by the top and bottom walls of the SIW substantially impacts the imaginary part of this parameter, which was taken into consideration in the proposed expression and was successfully used to represent Z_wave_ for SIWs of different widths up to 80 GHz. Furthermore, the model implementation based on the proposed expression can be applied to other SIW dimensions, provided that single-mode operation is maintained and the equivalence with a RWG remains valid. However, it is important to bear in mind that extending the application to higher frequencies necessitates verifying the appropriateness of the representation used for the series resistance, which must consider the specific metal surface profile required to keep metal losses within practical limits.

## Data Availability

The original contributions presented in the study are included in the article, further inquiries can be directed to the corresponding author.
